# Identifying maximal beta power from directional subthalamic local field potentials in Parkinson’s disease

**DOI:** 10.1038/s41531-026-01380-1

**Published:** 2026-05-08

**Authors:** Jennifer K. Behnke, Robert L. Peach, Moritz Gerster, Richard M. Köhler, Jeroen G. V. Habets, Johannes L. Busch, Varvara Mathiopoulou, Jonathan Kaplan, Lucia K. Feldmann, Juliette Vivien, Chi Wang Ip, Gerd-Helge Schneider, Katharina Faust, Patricia Krause, Andrea A. Kühn

**Affiliations:** 1 Movement Disorders and Neuromodulation Unit, Department of Neurology, Charité University Medicine, Berlin, Germany; 2 Berlin Institute of Health (BIH), Berlin, Germany; 3 Department of Neurology, University Hospital, Würzburg, Germany; 4 Department of Brain Sciences, Imperial College, London, UK; 5 UK Dementia Research Institute, Imperial College, London, UK; 6 Research Group Neural Interactions and Dynamics, Department of Neurology, Max Planck Institute for Human Cognitive and Brain Sciences, Leipzig, Germany; 7 Humboldt-Universität zu Berlin, Berlin School of Mind and Brain, Berlin, Germany; 8 Department of Neurosurgery, Charité University Medicine, Berlin, Germany; 9 Department of Neurosurgery, University Hospital, Düsseldorf, Germany; 10 NeuroCure Clinical Research Centre, Charité University Medicine, Berlin, Germany; 11 German Center for Neurodegenerative Diseases (DZNE), Berlin, Germany

**Keywords:** Neurology, Neuroscience

## Abstract

Accurate subthalamic beta activity could guide deep brain stimulation programming in Parkinson’s disease, but bipolar recordings complicate contact selection. In 39 patients, we validated three methods to estimate pseudo-monopolar beta power. Distance-weighted methods (Euclidean, Strelow) agreed consistently with the externalized “ground-truth” beta distribution. Maximal beta power across 20s windows was more stable in ring than in directional channels. Beta-contacts from all methods aligned with clinically active stimulation contacts one year after surgery.

Deep brain stimulation (DBS) is an effective treatment for advanced Parkinson’s disease (PD). Technological advancements, such as directional leads, have increased the complexity of DBS programming and tools for automated programming are urgently needed in clinical routine. In addition to imaging-based algorithms^1,2^, subthalamic (STN) beta activity (13-35 Hz) from local field potentials (LFPs) is a well-established biomarker for bradykinesia and rigidity in PD^3–7^, showing potential for guiding efficient DBS programming^8–10^. Current chronic sensing DBS devices record LFPs bipolarly from contact pairs on the same lead. The voltage difference is therefore measured between two contacts of the same lead, making it ambiguous to assign beta activity to a specific contact. To precisely guide electrophysiological programming, contact-wise estimates of beta activity are required, and pseudo-monopolar reconstruction methods have been developed to approximate the spatial distribution of beta power across individual contacts from bipolar recordings. This study addresses two key concerns of bipolar LFP sensing in DBS: i) how reliably can we estimate pseudo-monopolar beta power distributions across individual contacts that are critical for beta power-informed contact selection, and ii) how stable are beta power rankings across short recording durations of 21 s.

This study includes 39 PD patients implanted with bilateral subthalamic directional electrodes and Percept™ implantable pulse generators (IPGs; Medtronic, Minneapolis, MN, USA), who participated in at least one of two types of LFP recordings (Fig. 1a): shortly after lead implantation using externalized leads (*EXT*) and/or after IPG implantation (*IPG:* 12 months post-implantation) using 15 bipolar channels per lead (“BrainSense Survey” BSSU mode, Fig. 1b). *EXT* recordings provided longer recording durations of 2 min. *EXT-1* recordings used a common reference to provide high spatial resolution and served as a “ground truth” for monopolar estimations, while *EXT-2* simulated the bipolar IPG recording configuration by re-referencing the externalized LFPs (Fig. 1c). We validated and compared three methods for estimating pseudo-monopolar beta power distributions from *IPG* or *EXT-2* pseudo-IPG recordings. All three methods were previously described in Strelow et al.^11^ (Fig. 1f), Busch et al.^12^ (Fig. 1g) and Behnke et al.^13^ (*Euclidean method*, Fig. 1e) (details in the “Methods” section).

**Fig. 1:**
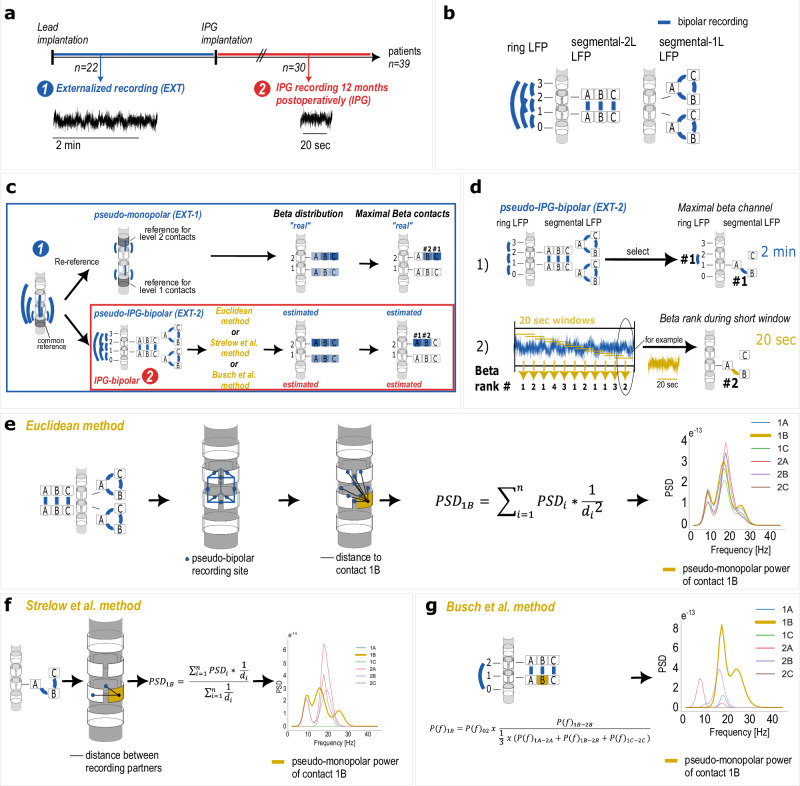
Methods. **a** Patient timeline consisting of at least one out of two LFP recordings. **b** IPG bipolar recording montage. LFPs were derived from DBS leads with eight contacts: ring contacts at levels 0 and 3 and segmented contacts at levels 1 and 2 (1 A/B/C, 2 A/B/C). IPG bipolar recording montage of 15 bipolar channels recorded in three channel groups: ring, segmental-2L and segmental-1L LFP. **c** LFP processing. Externalized LFPs (blue) were re-referenced in two formats: *EXT-1* with neighboring ring channels as reference for segmented channels, reflecting the most accurate “real” beta distribution from our externalized recordings; *EXT-2* was re-referenced to simulate the IPG bipolar recording montage. To estimate the beta distribution from IPG-bipolar recordings (red) and pseudo-IPG-bipolar *EXT-2* recordings (blue), three methods were applied. **d** Short-term stability of maximal beta power was analyzed by (1) selecting the maximal beta channel of 2-min *EXT-2* recordings (#1, separately for ring and segmental LFPs) and (2) extracting multiple 20-s windows with 50% overlap. Beta ranks (ranks #1 to #3 for ring channels and ranks #1 to #9 for segmental channels) were extracted from all short windows. **e** The Euclidean method utilizes all available *n* = 9 segmental LFPs weighted by the distance of their central position between the recording contacts and the contact of interest, for instance, 1B. **f** The Strelow et al. method utilizes *n* = 2 segmental-1L channels and weights them by the distance of the contact partners. **g** The Busch et al. method combines one ring LFP with the segmental-2L LFPs. Pseudo-monopolar power spectra from an example hemisphere are depicted on the right for each method.

When comparing estimated pseudo-monopolar beta distributions with the “real” EXT-1 recordings, all three methods showed significant, but variable correlations across hemispheres. The two distance-weighted approaches showed numerically higher correlation coefficients (Fig. 2a left panel, mean ± SD: *Euclidean* 0.44 ± 0.58, *n* = 28; Strelow et al. 0.41 ± 0.53, *n* = 28) than the Busch et al. method (0.36 ± 0.44, *n* = 28); however, no statistically significant differences were observed between the methods. When evaluating alignment with the “real” top-ranked beta contacts (#1, #2) in *EXT-1* in 28 hemispheres, the Euclidean method applied to *EXT-2* showed robust agreement beyond chance: 1) 75% (*p* = 0.124) included at least one top contact, 2) 54% (*p* < 0.0001) matched the #1 beta contact, and 3) 32% (*p* = 0.039) matched both contacts. The Strelow et al. method showed similar consistency: 1) 79%, *p* = 0.053; 2) 43%, *p* = *0.0028*; and 3) 29%, *p* = 0.122, while the Busch et al. method performed less reliably: 1) 71%, *p* = 0.251; 2) 32%, *p* = 0.086; and 3) 21%, *p* = 0.451 (Fig. 2a right panel).

**Fig. 2:**
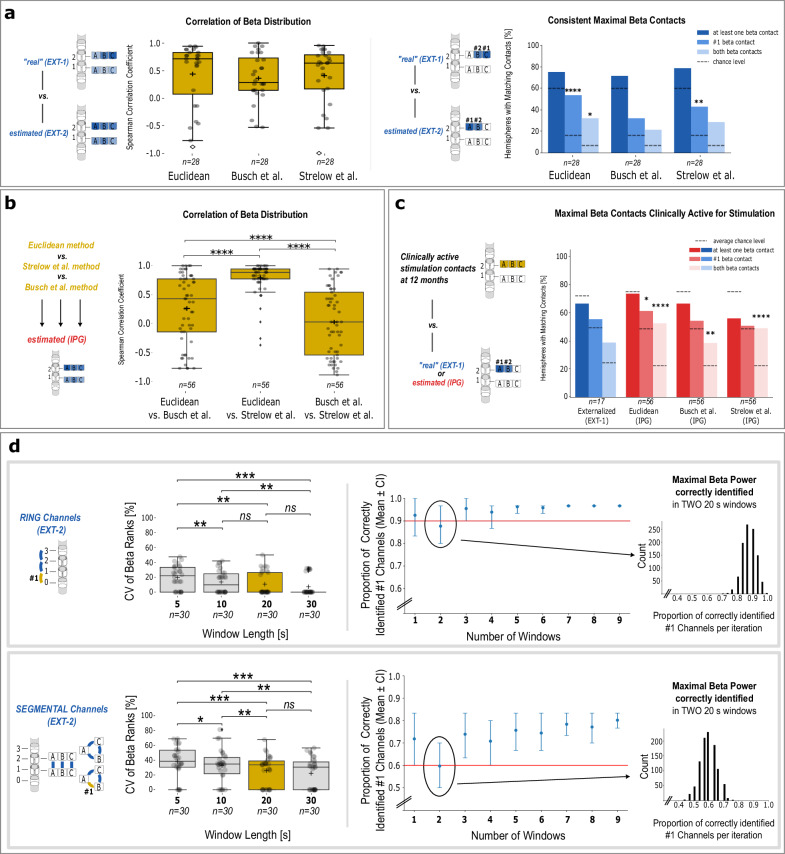
Results. **a** Validation of pseudo-monopolar power estimation methods. Estimated pseudo-monopolar beta distributions (left) and maximal beta contacts (#1, #2, right) using three methods (Euclidean, Busch et al., Strelow et al.) applied to *EXT-2* recordings were compared to the “real” *EXT-1* outcomes of the same hemispheres. Left: Most hemispheres showed positive correlation coefficients for all three methods. Right: Euclidean and Strelow et al. methods identified maximal beta contacts significantly more often than chance. **b** Agreement between methods. Estimated beta distributions using the Euclidean and Strelow et al. methods showed higher inter-method correlation than comparisons involving the Busch et al. method. **c** Agreement of maximal beta contacts with clinically active stimulation contacts. Maximal beta contacts derived from the “real” *EXT-1* distribution (blue bars) or from estimated distributions based on *IPG* recordings (red bars) were chronically active for stimulation at 12 months post-surgery in ~37–67% of hemispheres. **d** Short-term stability of beta ranks. Two-minute maximal beta channels were analyzed for ring (top) and segmental channels (bottom) from *n* = 30 leads (one additional hemisphere was excluded due to its recording being slightly shorter than 2 min). Left: Boxplots show that the coefficient of variance (CV) of beta ranks from 20-s windows was lower than from 5-s windows, and comparable to 30-s windows. In boxplots: black cross = group mean; horizontal line = median; vertical black line = data range; box = interquartile range; diamonds = outliers. Right: Proportion of #1 channels correctly identified from randomly selected 20-s windows across 1000 iterations. Each iteration involves a certain number of randomly selected windows (mean ± CI). An example is provided showing the proportions of correctly identified #1 channels from two selected windows. For ring channels, ≥3 windows ensured about 90% certainty (red line) in correctly identifying the #1 channel. For directional channels, ≥3 windows ensured 60% certainty.

We next compared these methods using *IPG* chronic recordings from 56 hemispheres. Estimated beta distributions generated by the Euclidean and the Strelow et al. methods correlated strongly with each other in most hemispheres (mean ± SD: 0.79 ± 0.28), while both showed weaker correlations with the Busch et al. method (Euclidean 0.27 ± 0.60, *p* < 0.0001; Strelow et al. 0.04 ± 0.58, *p* < 0.0001; Fig. 2b).

We further evaluated the temporal stability of beta power ranks using *EXT-2* LFPs, which simulate IPG recordings but offer a longer recording duration. To assess whether short recording sections (e.g., 20 s) are sufficient for identifying the top beta channel, we analyzed the variability of beta power ranks from channels showing maximal beta power averaged over 2 min across different window lengths (5, 10, 20, and 30 s). Two-minute maximal beta power was generally more stable for ring than segmental channels (Fig. 2d). As the available number of short windows per 2-min recording naturally decreased with increasing window length (5-s: *n* = 47, 10-s: *n* = 23, 20-s: *n* = *11*, 30-s: *n* = 7 windows per recording, Fig. 2d), we observed that 67% (20/30) of ring channels showed no change in the rank of the top beta channel (#1) across all 20-s windows, compared to only 33% (10/30) of segmental channels. The coefficient of variance in beta power ranks across windows was significantly lower for 20-s windows than 5-s windows (ring: 10.64 ± 15.61 vs. 19.70 ± 15.45, *p* = 0.003; segmental: 25.91 ± 20.10 vs. 38.23 ± 20.92, *p* = 0.0003), indicating higher temporal stability. No significant difference was observed between 20- and 30-s windows (ring: 7.20 ± 13.06, segmental: 22.22 ± 20.53, *p* > 0.05).

We then simulated how the number of short windows impacts the certainty of identifying maximal beta power. When randomly selecting 20-s windows from 2-min maximal beta channels, certainty of correctly identifying maximal beta power increased with more selected windows per iteration (*n* = 1000 *iterations*, Fig. 2d). For ring channels, certainty was around 80% when selecting only one window and increased to ≥90% with three windows (mean: 0.95, 95% CI: 0.90, 1.0) or more. Segmental channels were more variable: certainty was ≥60% when selecting only one window (mean: 0.72, 95% CI: 0.60, 0.83) and increased only slightly with additional windows.

To assess the clinical relevance of beta power measurements, we analyzed the overlap between estimated beta-ranked contacts and clinically active stimulation contacts at 12 months follow-up (FU) (Tables S2 and S3). Using *IPG* recordings (*n* = 56 hemispheres), alignment of the single top-ranked beta contact exceeded chance (mean chance: 48.8%) only for the Euclidean method (62.5%, *p* = 0.0137), but not for Strelow (48.8%, *p* = 0.312) and Busch methods (55.4%, *p* = 0.146). Additionally, alignment of both top-ranked beta contacts exceeded chance (mean chance: 22.3%) for all three methods (Euclidean 53.6%, *p* < 0.0001; Busch 39.3%, *p* = 0.003; Strelow 50.0%, *p* < 0.0001; see suppl. for further details, Table S3).

Altogether, we evaluated the usability of short bipolar beta power recordings as it is implemented in current devices such as the Percept for clinical contact selection in DBS. All tested methods for estimating pseudo-monopolar power from bipolar LFPs showed significant alignment with the actual beta distribution from *EXT-1* LFPs, but those incorporating power weighting that accounts for distances between recording contacts (Euclidean method and Strelow et al.) were overall superior. These differences underscore the complexity of bipolar LFPs that need to be considered for effectively implementing beta-guided automatized algorithms into clinical practice. We additionally assessed low-beta activity (13–20 Hz), and results followed overall a similar pattern to broadband beta (see suppl. for details, Fig. S1).

Furthermore, our data highlights that beta power fluctuations, especially in segmental channels, could impact beta-guided DBS programming algorithms^14,15^ when using short recordings. Using 2-min externalized LFPs, we demonstrated that ring channels exhibit greater stability of maximal beta power. Importantly, increasing the number of short window recordings improved the accuracy in determining maximal beta power, particularly for ring channels, but did not significantly differ when extending the recording time to 30-s windows. Further, regardless of the number of 20-s windows (one to nine), 2-min maximal beta power was correctly identified in more than 80% of ring channels (33% chance level) and 60% of segmental channels (11% chance level), with increasing accuracy for higher numbers of windows. An even number of windows can lead to ties in beta ranks, without a clear majority. Therefore, to reliably identify the maximal beta ring channel, we recommend repeating the 20-s recording once, and in case of a tie, repeating it once more (three recordings), which achieves over 90% certainty. Segmental channels show more fluctuations and need 9 repetitions to reach a certainty of around 80%.

Since beta power rankings were largely stable across short recording windows, discrepancies between segmented pseudo-monopolar beta estimates and the EXT-1 reference are unlikely to be driven by temporal instability. Instead, these differences likely reflect spatial factors inherent to bipolar sensing with directional leads, where recordings emphasize local voltage gradients. Variation between pseudo-monopolar estimation methods—particularly between approaches that do or do not incorporate distance-based power weighting—appears to primarily affect the spatial accuracy of segmented beta estimates.

Finally, estimated beta-based contact selection showed meaningful alignment with clinically selected stimulation contacts at 12 months post-surgery. Identification of the two top-ranked beta contacts consistently exceeded chance level for aligning with the clinical stimulation configuration, suggesting that pseudo-monopolar beta estimates preferentially identify clinically relevant stimulation sites. In contrast, early postoperative externalized recordings did not show above-chance performance, possibly due to beta suppression by the microlesional effect^16^ or minor shifts in lead rotation early after surgery^17^ (see suppl. for details), although interpretation is limited by the smaller sample size. These findings are in line with previous studies showing the potential of subthalamic beta oscillations to improve DBS programming efficiency^8,9,11,18^. A recent study introduced two new algorithms for predicting optimal contact levels from bipolar LFP recordings, achieving high accuracies for predicting the top two contact levels in a large multicenter cohort^19^. While that work focused on vertical contact levels, our study extends these efforts to the segmented contacts. In our cohort, directional stimulation was used in only 20% of hemispheres, possibly obscuring any potential directional fluctuations. This underutilization—likely due to its time-consuming programming^20^—further emphasizes the need for automated algorithms like beta-guided DBS programming for making directional stimulation programming more accessible in clinical practice.

## Limitations

To achieve high spatial resolution, referencing to a distant site (e.g., the contralateral DBS lead) can help isolate oscillatory activity at individual contacts. However, the choice of reference is critical. Ideally, it should exhibit minimal activity in the frequency band of interest, as strong signals at the reference (e.g., beta oscillations) can appear across all channels and obscure spatial differences. In this study, we recorded LFPs from externalized leads between segmented contacts and a common reference (the lowermost contact of one lead) for both leads. Using this recording montage, we chose to re-reference the LFPs, resulting in a sensing configuration between segmented contacts and their adjacent ring contacts, which achieved sufficient spatial resolution. While pseudo-monopolar beta power estimates showed significant correlations with EXT-1 reference data, the strength of these correlations was moderate and varied across hemispheres, reflecting the inherent ambiguity of reconstructing contact-wise power distributions from bipolar signals. This variability suggests that differences between estimation methods account for only part of the discrepancy in identifying maximal beta contact, and that additional factors likely contribute. Consequently, agreement rates of around 50–60% for identifying the single maximal beta contact, particularly for segmented contacts, indicate that pseudo-monopolar beta estimates should be interpreted as probabilistic guidance rather than definitive predictors in clinical application.

Short-term stability of maximal beta power was analyzed using 2-min externalized LFPs derived shortly after lead implantation. Therefore, the potential impact of microlesion effects contaminating LFPs should be considered. However, these recordings allowed for the required longer recording durations and common reference sensing configurations, which were fundamental for the analysis in this study. To assess the effect of repeated short recordings, the 2-min externalized recordings were segmented into multiple 20-s epochs, thereby simulating repeated short acquisitions. The suggested strategy of repeating multiple 20-s IPG recordings needs to be validated in future studies. Within the 2-min recording window, we do not expect other fluctuations (e.g., due to fluctuations of the dopaminergic state) as a confounder.

## Methods

39 PD patients (*n* = 12 female) with subthalamic bilateral B33005 “SenSight” directional electrodes (*n* = 78) and bidirectional Percept™ implantable pulse generators (IPGs; Medtronic, Minneapolis, MN, USA) were included (mean ± SD age 61 ± 8.96 years, disease duration 10 ± 4.49 years, MDS-UPDRS-III medication-off pre-surgery 51.89 ± 15.91, see Table S1). Surgeries were conducted at Charité—Universitätsmedizin Berlin as previously described^21^. Stimulation parameters were optimized during standard in-patient visits 3 and 12 months after surgery (Table S2). This study was approved by the local ethics committees (EA2/256/20) and adhered to the Declaration of Helsinki. All patients provided written informed consent. This patient cohort partially overlaps with a previously published dataset^13^, while the present study addresses distinct research questions. LFPs were recorded while participants were comfortably seated at rest with anti-Parkinsonian medication withdrawn for ≥12 h and stimulation switched off ≥30 min in advance^22^. Patients participated in at least one of two sessions (Fig. 1a):

1. *EXT*: shortly after lead implantation from externalized leads (*n* = 44 *hemispheres*)
2. *IPG*: 12 months post-IPG implantation (*n* = 60 *hemispheres*).

*EXT LFPs:* Externalized leads allowed for simultaneous recordings from eight channels per lead, with the lowermost contact of one lead as the common reference for both hemispheres for about 2 min duration. Recordings were amplified with a Saga64+ (Twente Medical Systems International, 4 kHz sampling rate). The last 2 min of *EXT LFPs* were down-sampled to 250 Hz, re-referenced to their ipsilateral lowermost contact, and processed into two formats (Fig. 1c, blue frame):

1. Pseudo-monopolar format *(EXT-1):* LFPs were re-referenced by subtracting segmental LFPs from the neighboring ring contact for high spatial resolution. This format offers a pseudo-ground truth for comparing the monopolar estimation methods.
2. Pseudo-IPG-bipolar format *(EXT-2):* LFPs were re-referenced to simulate the bipolar IPG channels (Fig. 1b). With this format, we can apply the pseudo-monopolar power estimation methods and compare outcomes with *EXT-1*. We can also test the stability of maximal beta power during short windows (5, 10, 20, and 30 s) with 50% overlap (Fig. 1d).

*IPG LFPs:* 20-s LFPs were derived from 15 bipolar channels per lead using the IPG (“BrainSense Survey” BSSU mode) with a sampling rate of 250 Hz and a high-pass filter at 1 Hz, in three separate groups (Fig. 1b). LFP recordings were exported to the JSON format for further offline analysis.

### Feature extraction

Spectrograms were computed using short-time Fourier transforms with 1-s Hanning windows and 25% overlap. Spectral beta power (13-35 Hz) was averaged over 2 min (*EXT-1, -2*) or 20 s (*IPG*). Periodic power was isolated using spectral parameterization (*specparam*)^23^ (details in supplementary materials). The “real” beta distribution was derived from *EXT-1* LFPs (Fig. 1c, blue frame), and the estimated beta distribution from bipolar *IPG*, or *EXT-2* LFPs (Fig. 1c, red frame), after applying three methods to estimate pseudo-monopolar power, previously described in Strelow et al^11^. (Fig. 1f), Busch et al.^12^ (Fig. 1g) and Behnke et al.^13^ (*Euclidean method*, Fig. 1e). Segmented contacts with maximal and second-highest beta power were labeled as #1 and #2, respectively. For the short-term stability analysis (Fig. 1d), notch-filtered (50 Hz) and band-pass filtered (5-95 Hz, Butterworth filter) power spectra were used without subtracting the aperiodic component. Hemispheres without a visible beta peak were excluded (*n* = 14 excluded for *EXT-1*, most likely due to the microlesional effect after surgery). A small number of hemispheres with EXT recordings were excluded because of insufficient beta power estimated across all pseudo-monopolar estimation methods, resulting in a common subset used for method comparison analyses (*n* = 28). All hemispheres (*n* = 60) of *IPG* showed a beta peak in at least one channel. However, for analyses involving clinically active stimulation contacts, four hemispheres with stimulation settings not suitable for analysis were excluded (Table S2).

### Behnke et al./Euclidean method^13^(Fig. 1e)

We developed a method that weighs spectral power based on the inverse squared Euclidean distance^24^ between each averaged recording site and the contact of interest. Details were previously described in Behnke et al.^13^. Briefly, this approach utilized all available bipolar LFPs from the segmented contacts (*n* = 9 LFPs). The sum of these weighted power spectra resulted in a single power spectrum for each segmented contact, as defined by Eq. (1):

$${{PSD}}_{1B}=\mathop{\sum }\limits_{i=1}^{n}{{PSD}}_{i}* \frac{1}{{d}_{i}2}$$

where *PSD*_*i*_ represents the spectral power of a bipolar channel *i*, and *d*_*i*_ is the Euclidean distance between the recording site (mean coordinates) and the contact of interest (e.g., 1B).

### Strelow et al. method^11^ (Fig. 1f)

Based on the method described in Strelow et al. we generated power spectra for segmented contacts only with two horizontal segmented channels (from the segmental-intra group), which include that segmented contact of interest as one of its two recording contacts. For instance, to obtain pseudo-monopolar power corresponding to contact 1B, the two channels 1A-1B and 1B-1C were used for the calculation. Both channels were weighted by dividing the spectrograms by the absolute distance in millimeters between the center of the contact of interest (e.g., contact 1B) and the other bipolar recording contact (e.g., contact 1 A and 1 C, respectively). Further, the weighted average spectrogram was generated by taking the sum of the weighted spectrograms and dividing them by the sum of the weights as summarized by Eq. (2)^11^. For more details, see Strelow et al.^11^:

$${{PSD}}_{1B}=\frac{{\sum }_{i=1}^{n}{{PSD}}_{i}* \,\frac{1}{{d}_{i}}}{{\sum }_{i=1}^{n}\frac{1}{{d}_{i}}}$$

In this formula *PSD*_*i*_ is the spectral power from the bipolar channel *i* of the *n* bipolar channels involving the segmented contact of interest. *d*_*i*_ is the distance between the two bipolar recording partners of the bipolar channel *i* in millimeters.

### Busch et al. method^12^ (Fig. 1g)

As described in Busch et al., power values of segmented contacts were estimated by combining the power spectra from a selected ring channel and multiplying it by the percentage spectrum of the vertical segmented channel (from the segmental-inter group) that includes that segmented contact. For segmented contacts in level 1 or 2, the ring channels 0-2 or 1-3 were chosen respectively, see Eq. (3). For more details, see Busch et al.^12^.

$${P(f)}_{1B}={P(f)}_{02}\,x\,\frac{{P(f)}_{1B-2B}}{\frac{1}{3}\,x\,({P\left(f\right)}_{1A-2A}+{P(f)}_{1B-2B}+{P(f)}_{1C-2C\,})}$$

### Statistics

1. *Pseudo-monopolar power estimation:* To compare pseudo-monopolar power estimations across methods, Spearman correlations were calculated between ranked beta distributions across directional contacts on the same lead. Fisher-transformed correlation coefficients were compared using paired t-tests on normally distributed data, or Wilcoxon signed-rank tests otherwise. Across all three methods, a Friedman test was used due to the non-normality of the data. Maximal beta contacts (#1, #2) were compared between the outcomes using methods for pseudomonopolar estimations and the *EXT-1* results, and consistency of contact selection was tested against chance using a binomial test. The chance levels were 60% for at least one contact, 16.7% for the #1 contact, and 6.7% for both contacts. To analyze the overlap of top-ranked beta contacts and clinically active stimulation contacts, hemisphere-specific chance probabilities were calculated based on the number of clinically active segmented contacts. Separate chance models assessed whether the beta #1 contact (chance EXT-1: 50.9%; IPG: 48.8%), both maximal beta rank #1 and #2 contacts (chance EXT-1: 25.9%; IPG: 22.3%), or at least one of the two beta-ranked contacts (chance EXT-1: 76.1%; IPG: 75.4%) overlapped with the clinically active configuration. Group-level significance was assessed using one-sided one-sample t-tests on the difference between observed outcomes and chance probabilities across hemispheres.
2. *Short-term reliability of maximal beta power:* The coefficient of variance (CV) for beta ranks from short windows was calculated from 2-min maximal beta channels (Fig. 1d) and compared between different window lengths using a Wilcoxon signed-rank test. A random selection of 20-s beta ranks from maximal beta channels was iterated 1000 times, and the proportion of channels correctly identifying maximal beta power was averaged (mean ± CI) across iterations. Ties between two ranks (e.g., #1 and #2) were not counted as correctly identifying maximal beta power.

Statistical significance was reported as: ns *p* ≥ 0.05, *p < 0.05, **p < 0.01, ***p < 0.001, ****p < 0.0001.

## Supplementary information

Supplementary information

## Data Availability

The data that support the findings of this study are available from the corresponding author upon reasonable request.

## Code availability

The underlying code for this study is available in the repository ‘monopolar_directional_beta’ and can be accessed via this link https://github.com/JKBehnke/monopolar_directional_beta.
