# Supplementary material for: Identifying maximal beta power from directional subthalamic local field potentials in Parkinson’s disease
Source: NPJ Parkinsons Dis. 2026 May 8;12:114. doi: 10.1038/s41531-026-01380-1 (PMC13156298; doi:10.1038/s41531-026-01380-1)
Supplement: Supplementary file 1 — Supplementary information [file 41531_2026_1380_MOESM1_ESM.pdf]

Identifying maximal beta power from directional subthalamic local field potentials in Parkinson's disease

Supplementary material

ID	Sex	Age (y)	Disease duration (y since diagnosis)	Clinical pheno-type	Symptom dominant side	MDS-UPDRS-III pre-surgery off/on	MDS-UPDRS-III 12 months post-surgery, med-off, stim-off/ on	Recording sessions	
**1**	f	70	11	M	right	49/30	44/16		IPG
**2**	f	43	2	A/R	left	36/11	19/13		IPG
**3**	f	71	13	TD	right	42/7	51/29		IPG
**4**	m	73	15	A/R	left	67/43	56/27	EXT	IPG
**5**	m	69	18	A/R	right	40/25	41/30	EXT	IPG
**6**	m	67	5	M	right	24/13	38/34		IPG
**7**	m	58	15	A/R	left	69/39	38/35	EXT	IPG
**8**	f	65	6	M	right	68/22	30/20	EXT	IPG
**9**	m	55	4	A/R	right	69/29	48/33	EXT	IPG
**10**	f	72	12	A/R	right	43/25	-	-	
**11**	m	45	7	A/R	right	76/33	-	EXT	
**12**	m	57	6	M	right	45/13	57/24		IPG
**13**	m	60	14	A/R	right	35/15	42/11		IPG
**14**	m	57	10	A/R	right	74/42	43/X		IPG
**15**	m	68	8	A/R	right	44/16	54/24		IPG
**16**	m	55	13	M	right	36/13	43/18		IPG
**17**	f	71	12	M	right	31/17	31/17	EXT	IPG
**18**	m	57	9	A/R	left	33/19	37/34	EXT	IPG
**19**	m	66	4	M	left	58/32	53/31	EXT	IPG
**20**	f	67	7	TD	left	51/23	40/28		IPG
**21**	m	53	15	A/R	right	64/27	52/32	EXT	IPG
**22**	m	61	6	M	right	33/15	40/27		IPG
**23**	f	63	11	A/R	left	36/15	-	EXT	
**24**	m	74	20	A/R	left	52/18	30/23	EXT	IPG
**25**	m	65	10	A/R	left	76/21	60/53	EXT	IPG
**26**	m	53	10	A/R	right	34/13	35/17	-	IPG
**27**	f	70	17	TD	right	52/10	49/32	-	IPG
**28**	m	52	13	M	left	34/18	-	EXT	
**29**	m	61	13	M	right	65/24	-		IPG
**30**	f	66	9	M	left	50/26	-	EXT	
**31**	f	64	5	A/R	right	31/13	-	EXT	IPG

**32**	m	51	10	M	right	90/55	-	EXT	
**33**	m	70	6	M	right	73/36	-	EXT	IPG
**34**	m	68	9	A/R	both	40/30	-	EXT	IPG
**35**	m	42	21	M	left	90/42	-	EXT	
**36**	f	56	10	M	left	59/27	-	EXT	
**37**	m	44	11	A/R	left	52/30	-	EXT	
**38**	m	57	15	A/R	left	58/39	-		IPG
**39**	m	75	12	M	right	49/24	-		IPG

Table S1: Clinical and demographic details from 39 PD patients (mean ± SD age 61 ± 8.9 years, disease duration 10 ± 4.5 years, MDS-UPDRS-III medication-off before surgery 51.9 ± 15.9). LFP recordings were performed from externalized leads (EXT, *n=44*), and from the IPG 12 months after surgery (IPG, *n=60*). f = female, m = male, M = mixed, TD = tremor-dominant, A/R = akinetic-rigid PD phenotype, y = years.

ID	Stimulation contacts, amplitude LSTN	Stimulation contacts, amplitude RSTN	Stimulation pulse width, frequency	Contact with maximal pseudo-monopolar beta power LSTN / RSTN
**1**	2- 3-, 1.05 mA	2- 3-, 1.05 mA	40 µs, 100 Hz	1B / 3
**2**	1-, 1.5 mA	0-, 1.5 mA	60 µs, 130 Hz	1A / 1A
**3**	0- **1B-**, 1.9 mA	0- **1B-**, 2.1 mA	60 µs, 130 Hz	1C / 1A
**4**	1-, 4.7 mA	1-, 4.3 mA	40 µs, 90 Hz	1A / 2A
**5**	2-, 2.5 mA	2-, 2.3 mA	60 µs, 125 Hz	1A / 2A
**6**	**1A- 1B-**, 5.1 mA	**2A- 2B-**, 4.5 mA	60 µs, 125 Hz	1A / 1A
**7**	**1C- 2C-**, 2.5 mA	1-, 2.6 mA	60 µs, 125 Hz	1A / 2A
**8**	**2C-**, 0.9 mA	**1C- 2C-**, 1.4 mA	60 µs, 125 Hz	1A / 2A
**9**	2-, 2.8 mA	2-, 2.8 mA	60 µs, 130 Hz	1C / 3
**12**	**2A- 2B-** 3-, 2.8 mA	**2A- 2B-** 3-, 3.1 mA	60 µs, 125 Hz	2A / 2B
**13**	1- 2-, 2.3 mA	1- 2-, 2.3 mA	60 µs, 100 Hz	1C / 1C
**14**	2-, 3,4 mA	2-, 2.7 mA	60 µs, 130 Hz	2B / 1A
**15**	2-, 2 mA	1-, 2 mA	60 µs, 110 Hz	3 / 1B
**16**	1- 3-, 1 mA	2-, 1.8 mA	60 µs, 110 Hz	1A / 2A
**17**	1- 2-, 1.5 mA	1- 2-, 1.2 mA	60 µs, 90 Hz	2C / 2C
**18**	1-, 2.8 mA	0-, 2.8 mA	40 µs, 130 Hz	1B / 1B
**19**	1-, 1 mA	1-, 3 mA	60 µs, 130 Hz	2A / 0
**20**	**2B- 2C-** 3-, 2.3 mA	**2B- 2C-** 3-, 2.6 mA	60 µs, 110 Hz	1A / 1B
**21**	1+ 2- 3+, 2.5 mA	**1B- 2B-**, 5.7 mA	60 µs, 130 Hz	2C / 2B
**22**	1-, 2.5 mA	1-, 2.5 mA	60 µs, 100 Hz	2C / 1C
**24**	1-, 2.2 mA	2-, 2.6 mA	60 µs, 125 Hz	0 / 1B
**25**	2-, 2.7 mA	2-, 2.7 mA	60 µs, 90 Hz	2A / 2A
**26**	1-, 2.4 mA	1-, 2.3 mA	60 µs, 85 Hz	2A / 2A
**27**	2- 3-, 1.9 mA	2- 3-, 1.7 mA	60 µs, 100 Hz	2A / 2A
**29**	1-, 1.7 mA	1-, 2 mA	60 µs, 125 Hz	1A / 1B
**31**	3-, 1.4 mA	2A-, 2B-, 0.9 mA	60 µs, 130 Hz	2C / 2B
**33**	2-, 2.8 mA	2-, 2.6 mA	60 µs, 180 Hz	2C / 2A
**34**	1-, 1.8 mA	1-, 1.8 mA	60 µs, 130 Hz	2A / 1C
**38**	2-, 1.5 mA	2-, 1.5 mA	60 µs, 130 Hz	2C / 2B
**39**	1-, 2.6 mA	2-, 2.3 mA	60 µs, 130 Hz	1C / 2A

Table S2. Stimulation parameters and contacts with maximal pseudo-monopolar beta power using the Euclidean method at 12 months post-surgery. For hemispheres with directional stimulation, active contacts are written in bold. If no contact in the two middle levels was active for chronic stimulation or a bipolar stimulation setting was used, the contacts are in gray color and corresponding hemispheres were excluded from the analysis (*n=4* hemispheres). Directional stimulation was used in 12 of 60 hemispheres.

***specparam* parameters:** (1)

Externalized LFPs:

- Frequency range 2-45 Hz (because of the plateau at higher frequencies)
- peak_width_limits = [3, 20.0], max_n_peaks = 3, min_peak_height = 0.1, aperiodic_mode = "fixed".
- DBS leads: 44
- Total number of fitted channels: 308 (6 directional contacts and uppermost ring contact per lead)
- Specparam error mean ± SEM: 0.069 ± 0.002
- Specparam r squared mean ± SEM: 0.9501 ± 0.003

IPG-12 LFPs:

- Frequency range 2-95 Hz
- peak_width_limits = [3, 20.0], max_n_peaks = 4, min_peak_height = 0.1, aperiodic_mode = "fixed".
- DBS leads: 60
- Total number of fitted channels: 720 (12 bipolar channels per lead)
- Specparam error mean ± SEM: 0.075 ± 0.0005
- Specparam r squared mean ± SEM: 0.974 ± 0.0006

Power spectra were fitted without respecting a knee in the aperiodic component, since recent studies have shown the absence of a knee in the aperiodic component in the STN (2).

Agreement of maximal beta contacts with clinically active stimulation contacts

At least one of the two top-ranked beta contacts (#1, #2) overlapped with clinically active stimulation contacts in 75.0 %, 51.8 %, and 67.9 % of hemispheres (*n=56* hemispheres) for the Euclidean, Strelow, and Busch methods, respectively. This did not exceed hemisphere-specific chance levels (mean chance across hemispheres: 75.4 %). Beta-guided contact selection based on EXT-1 recordings (n=17) did not exceed chance (both beta contacts: 41.2 %; #1 beta contact: 58.8 %; at least one beta contact: 70.6 %).

**Method**	At least one	Beta #1	Beta #1 and #2
**Euclidean IPG**	75.0 %	**62.5 %** *	**53.6 %** ****
**Busch IPG**	67.9 %	55.4 %	**39.3 %** **
**Strelow IPG**	57.1 %	51.8 %	**50.0 %** ****
n hemispheres	52	52	52
Chance (mean)	75.4 %	48.8 %	22.3 %
**Externalized EXT-1**	70.6 %	58.8 %	41.2 %
n hemispheres	28	28	28
Chance (mean)	76.1 %	50.9 %	25.9 %

Table S3. Agreement of maximal beta contacts with clinically active stimulation contacts. Percentage of observed alignment of at least one of the top two beta-ranked contacts, single top-ranked beta contact (#1,) or two top beta-ranked contacts (#1, #2) derived from the three pseudo-monopolar estimation methods (Euclidean, Strelow, Busch) from IPG recordings (top) and from externalized (EXT-1) recordings (bottom). Significance for observed alignment of beta-contacts with clinically active contacts above the mean of hemisphere-specific chance probabilities across hemispheres are highlighted with stars and written in bold. Chance levels between the IPG and EXT-1 recordings differ because of different sample sizes.

Low-beta (13-20 Hz) analysis

To assess whether restricting the analysis to low-beta activity (13–20 Hz) affects method performance, all analyses were repeated using low-beta power (Supplementary Fig. S1). Overall, low-beta based results followed a similar pattern to broadband beta. However, low-beta peaks were less consistently detectable across hemispheres in externalized recordings, resulting in reduced sample sizes and increased variability. Therefore, broadband beta power (13-35 Hz), which is widely used as robust biomarker of motor state and treatment effects, was used for the primary analyses, as restricting the analysis to low-beta did not markedly improve agreement between pseudo-monopolar estimation methods or overall robustness. While low-beta may offer complementary information, broadband beta provided more consistent signal availability across hemispheres in this

cohort. Alternative measures such as peak amplitude or the aperiodic component may offer additional physiological specificity and should be addressed in future studies.

In the subset of 21 hemispheres, which exhibited a low-beta peak across all methods, correlations between estimated (EXT-2) and “real” (EXT-1) low-beta distributions were moderate and variable (Euclidean: mean r=0.37; Strelow et al.: 0.20; Busch et al.: 0.31), with pairwise comparisons showing significantly higher correlations for the Euclidean method compared to the Strelow et al. method (Wilcoxon signed-rank test, p = 0.0019), while other pairwise differences were not significant. A global Friedman test across methods did not reach significance (p=0.053). Agreement between estimated and “real” maximal low-beta contacts in EXT recordings exceeded chance only for the Euclidean method, both for identifying the single top-ranked contact (#1: 38.1%, p=0.016) and both top-ranked contacts (#1 and #2: 28.6%, p=0.002). Neither the Busch nor the Strelow et al. methods exceeded chance for any of the low-beta contact-ranking criteria in EXT recordings.

In IPG recordings, all 56 hemispheres showed a low-beta peak in at least one channel. Correlations between low-beta distributions estimated by different methods followed a similar pattern to broadband beta, with strong agreement between the Euclidean and Strelow et al. methods (mean r=0.74) and weaker agreement involving the Busch et al. method (mean r Busch et al.: 0.05; Euclidean: 0.30), although overall correlation coefficients were lower than those observed for broadband beta (all pairwise comparisons p < 0.001).

With respect to clinical relevance, alignment between clinically active contacts and the single top-ranked low-beta contact exceeded chance for the Euclidean (62.5%, p=0.015) and Busch et al. (60.7%, p=0.032) methods, while alignment of both top-ranked contacts exceeded chance for all three methods (Euclidean: 51.8%, p<0.00001; Busch: 42.9%, p=0.0008; Strelow et al.: 53.6%, p<0.00001). Alignment of at least one of the two top-ranked low-beta contacts did not exceed chance for any method. Low-beta based

alignment using externalized recordings (n=9 hemispheres) did not robustly exceed chance for any criterion.

Overall, restricting the analysis to low-beta power yielded patterns similar to broadband beta across method comparisons and clinical alignment. However, low-beta correlations were generally weaker and less consistently detectable across hemispheres, whereas broadband beta provided more robust signal availability, particularly in externalized recordings.

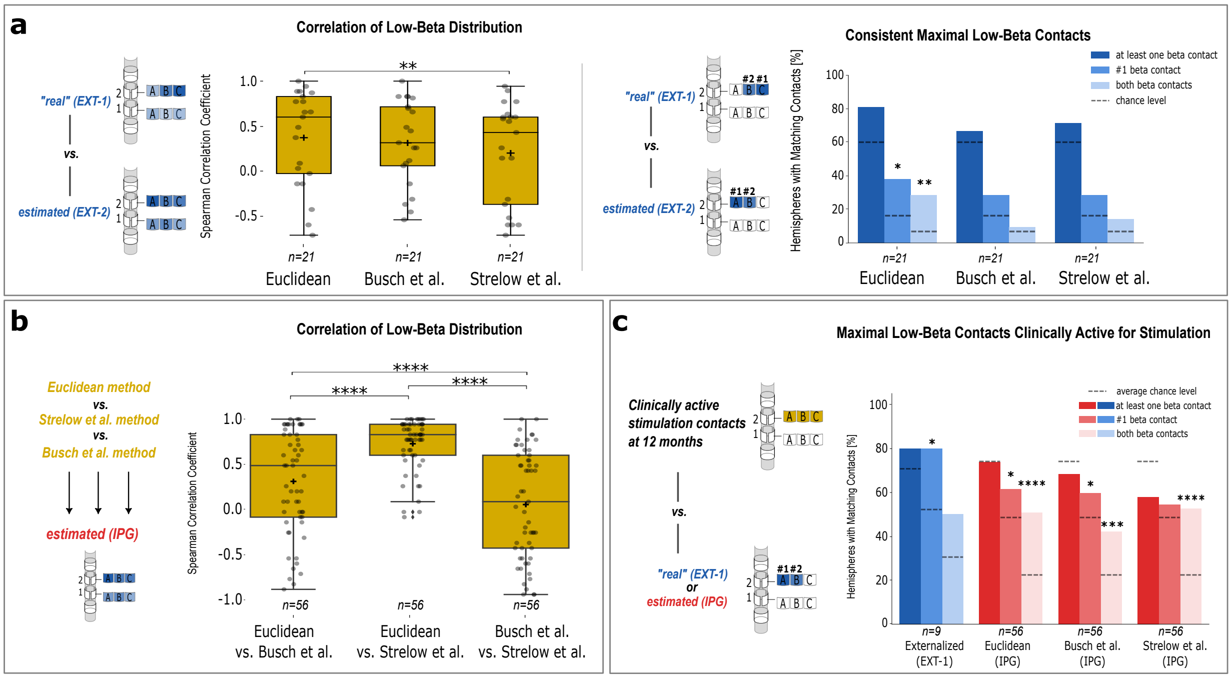

Figure S1. Low-beta (13–20 Hz) analysis of pseudo-monopolar power estimation methods. a, Validation of pseudo-monopolar low-beta power estimation methods. Estimated pseudo-monopolar low-beta distributions (left) and maximal low-beta contacts (#1, #2; right) obtained using three methods (Euclidean, Busch et al., Strelow et al.) applied to EXT-2 recordings were compared with the corresponding "real" EXT-1 low-beta distributions of the same hemispheres. Left: Correlation coefficients between estimated and real low-beta distributions were moderate and variable across hemispheres, with overall lower values compared to broadband beta. Right: Only the Euclidean method identified maximal low-beta contacts significantly exceeding chance. **b, Agreement between methods**. Low-beta distributions estimated using the Euclidean

and Strelow et al. methods showed higher inter-method correlation than comparisons involving the Busch et al. method. **c, Alignment of maximal low-beta contacts with clinically active stimulation contacts.** Using IPG recordings, alignment between clinically active contacts and both top-ranked low-beta contacts exceeded chance for all three methods, while alignment with the single top-ranked low-beta contact exceeded chance for the Euclidean and Busch et al. methods.

Lead rotation and beta power localization anatomically within the STN

When comparing early externalized recordings to clinically active contacts at 12 months we did not account for potential small changes in the rotational orientation of directional leads over time. Although previous studies suggest that substantial postoperative rotation is uncommon (3), subtle shifts cannot be excluded and could influence segmented beta distributions. Lastly, an important avenue for future work will be to relate bipolar and pseudo-monopolar beta distributions to individual lead localizations and anatomically defined sweet spots. In this study, we focused on relative beta rankings within each lead and did not spatially normalize electrode positions anatomically within the STN.

References

1. Donoghue T, Haller M, Peterson EJ, Varma P, Sebastian P, Gao R, et al. Parameterizing neural power spectra into periodic and aperiodic components. Nat Neurosci. 2020;23(12):1655-65.
2. Bush A, Zou J, Lipski WJ, Kokkinos V, Richardson RM. Broadband aperiodic components of local field potentials reflect inherent differences between cortical and subcortical activity. bioRxiv. 2023.
3. Cavalloni F, Brugger F, Kagi G, Naseri Y, Brogle D, Bozinov O, et al. Evaluation of the Rotational Stability of Directional Deep Brain Stimulation Leads: A Case Series and Systematic Review. J Neurol Surg A Cent Eur Neurosurg. 2024;85(3):288-93.
